# Lack of Dietary Polyunsaturated Fatty Acids Causes Synapse Dysfunction in the *Drosophila* Visual System

**DOI:** 10.1371/journal.pone.0135353

**Published:** 2015-08-26

**Authors:** Anna B. Ziegler, Cindy Ménagé, Stéphane Grégoire, Thibault Garcia, Jean-François Ferveur, Lionel Bretillon, Yael Grosjean

**Affiliations:** 1 CNRS, UMR6265 CSGA, 21000, Dijon, France; 2 INRA, UMR1324 CSGA, 21000, Dijon, France; 3 Université de Bourgogne-Franche-Comté, UMR CSGA, 21000, Dijon, France; Ecole Normale Supérieure, FRANCE

## Abstract

Polyunsaturated fatty acids (PUFAs) are essential nutrients for animals and necessary for the normal functioning of the nervous system. A lack of PUFAs can result from the consumption of a deficient diet or genetic factors, which impact PUFA uptake and metabolism. Both can cause synaptic dysfunction, which is associated with numerous disorders. However, there is a knowledge gap linking these neuronal dysfunctions and their underlying molecular mechanisms. Because of its genetic manipulability and its easy, fast, and cheap breeding, *Drosophila melanogaster* has emerged as an excellent model organism for genetic screens, helping to identify the genetic bases of such events. As a first step towards the understanding of PUFA implications in *Drosophila* synaptic physiology we designed a breeding medium containing only very low amounts of PUFAs. We then used the fly’s visual system, a well-established model for studying signal transmission and neurological disorders, to measure the effects of a PUFA deficiency on synaptic function. Using both visual performance and eye electrophysiology, we found that PUFA deficiency strongly affected synaptic transmission in the fly’s visual system. These defects were rescued by diets containing omega-3 or omega-6 PUFAs alone or in combination. In summary, manipulating PUFA contents in the fly’s diet was powerful to investigate the role of these nutrients on the fly´s visual synaptic function. This study aims at showing how the first visual synapse of *Drosophila* can serve as a simple model to study the effects of PUFAs on synapse function. A similar approach could be further used to screen for genetic factors underlying the molecular mechanisms of synaptic dysfunctions associated with altered PUFA levels.

## Introduction

For most animals, polyunsaturated fatty acids (PUFAs) are essential nutrients. Essential PUFAs make up about 20% of the dry weight of the lipid-rich nervous system [1]. The effects of chronic deficiency in dietary PUFAs or genetically determined abnormalities in lipid metabolism are under investigation in order to understand the precise role of PUFAs in the functioning of the brain [2–4]. It is well accepted that PUFAs have an important impact on synapse function [5–7]. Their absence could lead to neurotransmission malfunction, which might promote the onset or progression of specific neurological diseases [8–10].

Most circulating fatty acids (FAs) are derived from the food, at least in animals with a mixed diet [11,12]. FAs are mostly incorporated after being hydrolysed from Triacylgycerol (TAG) in the gastric and intestinal lumen. They probably reach the brain via simple diffusion and protein mediated transport. Once transported into brain cells those FAs can be activated and acetylated to a glycerol backbone so as to form Diacylglycerol (DAGs) or TAGs. Therefore, not only the amount of free PUFAs but also the amount of PUFAs bound in DAGs and TAGs in the brain might depend on the ingested food.

Several studies have suggested a relationship between low omega-3 PUFA dietary consumption and a greater risk of neurological impairments, including depression. Inversely high omega-3 PUFA intake reduces the risk for age-related macular degeneration [13–15].

PUFA deficiency can be caused not only by malnutrition but also by genetic factors that impact the uptake and metabolism of PUFAs. For example, several studies have pointed out a correlation between depressive disorders and genetically defined low activity of type VI phospholipase A_2_ and fatty acid CoA ligase 4, both leading to decreased omega-3 PUFA levels [3,16,17]. However, detailed models explaining the precise role of PUFAs in synapse function as well as the link to the onset and progression of neurological diseases are still missing.

The nervous system and fatty acid metabolism of the fruit fly *Drosophila melanogaster* share many important characteristics with those of vertebrates. Also, the *Drosophila’s* genetic versatility, its rapid life cycle, and the large numbers of individuals that can be generated in a limited space make this model organism ideal for the sophisticated genetic screens necessary to discover associations between PUFAs and specific neuronal functions.

As most eukaryotes, *Drosophila* are capable of *de novo* synthesis of non-essential saturated fatty acids (SFA) or mono-unsaturated fatty acids (MUFA) through the activity of acetyl-CoA carboxylase and fatty acid synthetase yielding the saturated 16-carbon long palmitic acid (C16:0). Elongase enzymes can add two carbons to this substrate to produce stearic acid (C18:0); and fatty acid desaturase enzymes can introduce a double bond to create mono-unsaturated fatty acids such as palmitoleic acid (C16:1) or oleic acid (C18:1) [18–21].

Like most animals, fruit flies lack enzymes that allow the production of the essential omega-6 PUFA alpha-linoleic acid (C18:2) and the essential omega-3 PUFA alpha-linolenic acid (C18:3). Thus, they need to find them in their diet. Moreover, C18:2 and C18:3 are the most abundant PUFAs in *Drosophila*. When fruit flies incorporate long-chain fatty acids such as adrenic acid (C22:4) or docosahexaenoic acid (C22:6) in their diet, those FA are broken down into arachidonic acid (C20:4) or eicosapentaenoic acid (C20:5). Thus, in contrast to mammals, *Drosophila* lack long-chain fatty acids with chains longer than 20 carbons [21–23].

To study the impact of PUFA starvation on neurotransmission we aimed at developing a fly diet that contained only very low amounts of PUFAs thus causing a severe PUFA deficiency. As a model of synaptic transmission in *Drosophila*, we used the fly´s visual system.

## Material and Methods

### Fly Stocks and Food Preparation

*Drosophila melanogaster* of the Canton Special (CantonS) strain were cultured at 25°C with a 12-hour light, 12-hour dark cycle on our regular lab food. Our regular lab food is composed of 6.5% yeast (Type E 50, lipid contend in dry mass: 2%; Sensient Flavors, Strasbourg, France), 6.5% corn meal (Eurogerm, Saint-Apollinaire, France), 1% agar-agar (Kalys Gastronomie, Bernin, France) and 0.5% Methyl 4-Hydroxybenzoate (Prolabo). PUFA-free food lacked corn meal and contained 17% yeast, 1% agar-agar and 10% crystal sugar (Beghin Say, Lille, France) to adjust the level of calories. To test the effect of specific fatty acids, each one was added to this PUFA-free medium at the following concentration: 5 mM stearic acid (18:0, Ref. 9459.1 Carl Roth), 2.5 mM oleic acid (18:1, Ref. 14429J, INTERCHIM), 5.7 mM linoleic acid (C18:2 omega-6, Ref. 13558X INTERCHIM), 2.3 mM linolenic acid (C18:3 omega-3, Ref. 09072H INTERCHIM), 25.1 μl/ml walnut oil, and 4,8 μl/ml linseed oil (Bio Planete, Bram, France), as previously done [24,25]. The detailed composition of the walnut oil and linseed oil can be seen in S1 Table. To insure adequate intake in vitamin A, 0.4 μM of beta-Carotene [26] (22040 Sigma-Aldrich) was added to each medium.

### Positive Phototaxis Assay

Positive phototaxis was measured as described by Benzer, 1967 [27]. Briefly, a countercurrent apparatus was used to fractionate a population of 20–25 naïve CantonS male flies (2–9 days old). To avoid variation in performance linked to circadian rhythm, experiments were carried out between 1 and 3 hours after lights on (corresponding to 8:00–10:00 AM). Experiments were always carried out in the same room at 25°C. All flies of one group started the first trial in the same vial (vial 0). They were forced to the bottom of the vial, the apparatus was placed horizontally, and the flies were given the chance to walk 11.5 cm towards a light source within seven seconds (18W cool white, Sylvania Luxline Plus F18W). This duration was chosen based on preliminary experiments to allow our diet manipulation either to increase or decrease the behavioral performance. Flies, which walked towards the light source, were shifted to the next vial (vial 1). This procedure was repeated 5 times in total. At the end, all flies were distributed into vials 0 to 5 depending on how often they walked towards the light source. We calculated a phototaxis index ∑(i*N_i_)/N with N representing the total number of flies assayed, i the tube number (0–5), and Ni the number of flies in the i^th^ tube at the end of the experiment.

### Electroretinographic Recordings

ERGs were obtained and evaluated as described recently [28,29]. Briefly, 3- to 7-day-old male CantonS flies were immobilized in a cut-off tip. The indifferent electrode was placed in the proboscis while the recording electrode was placed in contact with the eye. After 5 minutes of dark adaptation, a stable baseline was obtained and a 1 s light stimulus was applied. ERG plots were obtained by calculating the mean of 10 light stimuli per fly. The sustained negative potential (SNP) was defined by the lowest value prior to the “off”-transient, the “on”-transient by the highest value after the onset of the light stimulus. The “off”-transient was defined by subtracting the lowest value after the offset of light from the sustained negative potential.

### Locomotor Activity Test

Locomotor activity was assayed between 1 and 3 hours after lights on. Single 2- to 9- day-old *Drosophila* male flies were introduced into a chamber created by placing a watch glass (3.5 cm^2^) on a glass plate using an aspirator [30]. The chamber was positioned under a digital monochrome camera (Bosch LTC0355/50) and locomotor activity was automatically recorded for 10 minutes with 5 frames per second. Data acquisition and analysis were automatically performed using EthoVision software (Noldus, Wageningen, Netherlands).

### Lipid Analyses

Lipids were extracted from *Drosophila* heads (3 pools of 30 heads) according to the Folch method and submitted to transmethylation of the fatty acids using boron trifluoride in methanol according to Morrison and Smith [31,32]. Fatty acid methyl esters were subsequently extracted with hexane and analyzed using gas chromatography on a Hewlett Packard Model 5890 gas chromatograph (Palo Alto, CA, USA) using a CPSIL-88 column (100 m × 0.25 mm i.d., 0.20 μm film thickness, Varian, Les Ulis, France) equipped with a flame ionization detector. Hydrogen was used as the carrier gas (inlet pressure, 210 kPa). The oven temperature was held at 60°C for 5 min, increased to 165°C at 15°C/min and held for 1 min and then to 225 C at 2°C/min and finally held at 225°C for 17 min. The injector and the detector were maintained at 250°C. Fatty acid methyl esters were identified by comparison to commercial and synthetic standards (Sigma Aldrich, L’Isle d’Abeau, France). The data were processed using the EZChrom Elite software (Agilent Technologies, Massy, France) and reported as a percentage of the total fatty acids.

### Data Analysis and Statistics

All data were transferred to Prism 5.0d (GraphPad) for statistical analysis and tested for normal distribution using the D´Agostino and Pearson omnibus normality test.

Two pairs of data (for example between data obtained from flies raised on low-PUFA diet and on high PUFA diet) were analyzed using the paired-t-test if they had passed the normality test. Pairs of data, which did not pass the normality test, were analyzed using the Mann-Whitney test.

Groups of more than 2 data sets, which were normally distributed, (for example flies raised on low-PUFA diet, on high-PUFA diet, and high-PUFA+18:0) were compared with the one-way analysis of variance (one-way ANOVA) test followed by a Bonferroni’s post-hoc test. Groups of multi-data sets, which were not normally distributed, were analyzed by a Kruskal-Wallis (KW) test followed by a Dunn’s post-hoc test. Data sets with N ≤ 10 were analyzed using non-parametric statistical tests.

## Results

### Fatty Acid Composition of Drosophila Head Is Altered by the Diet

**Fig 1:**
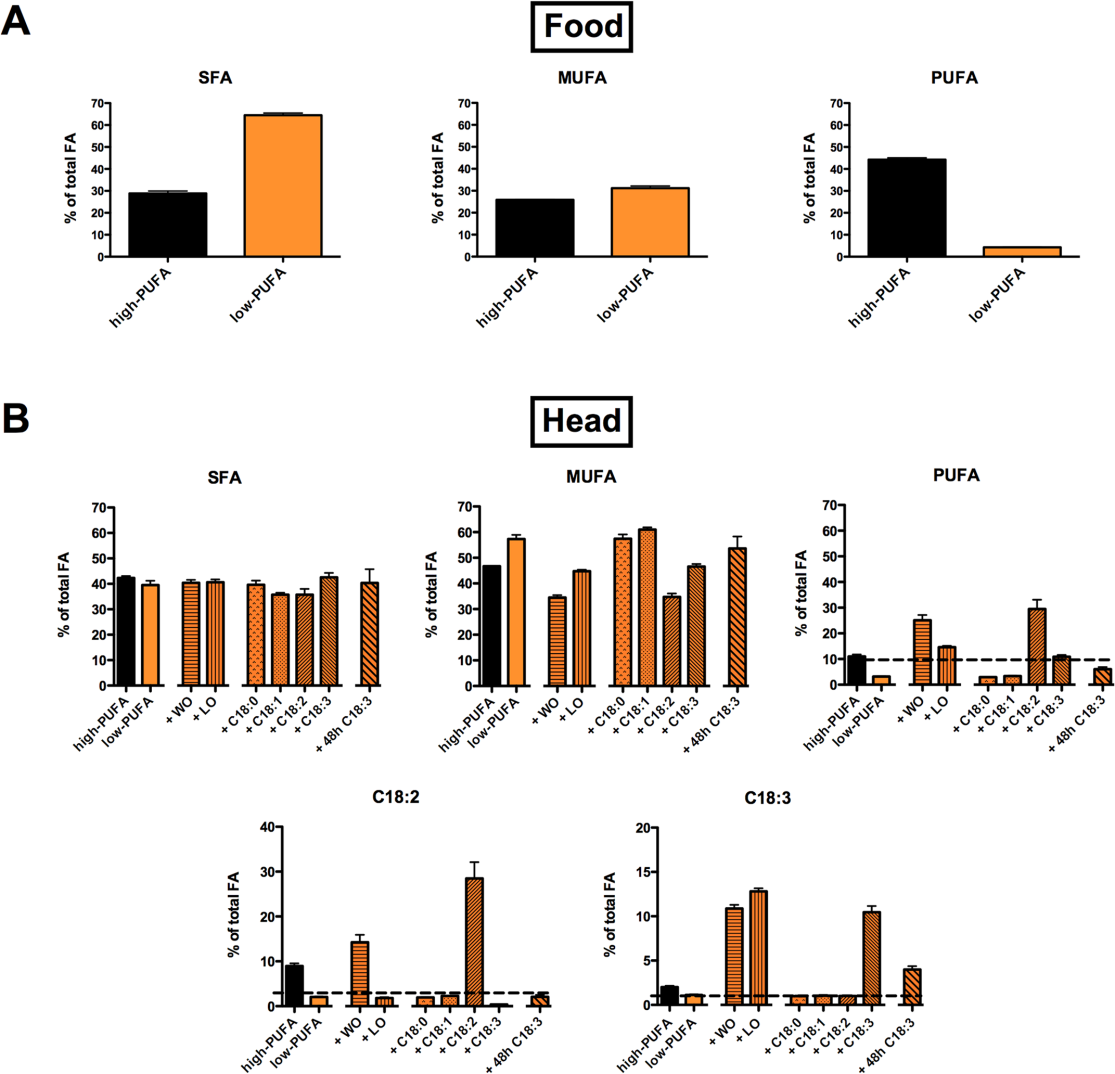
Fatty acid contents in fly food and fly heads. (A) relative amounts of saturated fatty acids (SFA), mono-unsaturated fatty acids (MUFA), and poly-unsaturated fatty acids (PUFA) in two *Drosophila* diets (high-PUFA diet in black, low-PUFA diet in orange) (N = 4). (B) relative amounts of SFA, MUFA, and PUFA in *Drosophila* heads (N = 3 groups of 30 heads). FA contents are indicated in black when flies were raised on a high-PUFA diet or orange when flies were raised on a low-PUFA diet. Bars in orange and black indicate that flies were raised on low-PUFA diet enriched with walnut oil (WO) or linseed oil (LO), which both contain non-essential and essential FA, single non-essential fatty acid (C18:0 or C18:1) or single essential fatty acids (C18:2 or C18:3). The orange and black diagonally striped bar on the very right indicates FA contents of fly heads when animals were raised on a low-PUFA diet and then were fed with a low-PUFA diet enriched in C18:3 for 48 hours during their adulthood. A complete FA analysis and absolute values can be observed in SF1, SF2, and ST2. Data in B represent mean ± SEM.

The aim of this study was to assess the consequences of a lack of dietary polyunsaturated fatty acids on the neuronal function in the fruit fly *Drosophila melanogaster*. Therefore, we first developed a *Drosophila* diet with a very low PUFA content. Hereafter it is referred to as “low-PUFA diet”. This diet lacked maize, which is the main source of PUFAs in our regular laboratory *Drosophila* food. We analyzed the fatty acid composition of this diet using gas chromatography (GC), and compared it to our regular lab food, which we called “high-PUFA diet” (Fig 1A, SF1). Although our low-PUFA food had a fair amount of non-essential saturated and mono-unsaturated fatty acids, the absolute amount of PUFAs was reduced by more than 90% compared to the high-PUFA diet (Fig 1A).

We next measured to what extent the FAs present in the fly reflect the FAs present in their diet. For this experiment only fly heads, mostly consisting of eyes and brains, were used. We found that animals raised on the low-PUFA food had similar amounts of SFAs and MUFAs in their heads as did flies fed with the high-PUFA diet, but their PUFA content was reduced by 72% (Fig 1B). We then analyzed the change of the most abundant PUFAs in *Drosophila* heads, which were omega-6 linoleic acid (C18:2) and omega-3 linolenic acid (C18:3). The relative amount of C18:2 dropped by 80% and the relative amount of C18:3 by 50% in fly heads when the animals were bred on a low-PUFA diet (Fig 1B, see also S2 Fig and S1 Table for absolute values).

Next, we used two different methods to recover the PUFA contents in the heads of flies raised on low-PUFA food. First, we added walnut oil or linseed oil to the low-PUFA diet. These oils are respectively rich in linolenic and linoleic acids (S1 Table). Walnut oil, which contains high amounts of C18:2 and low amounts of C18:3, induced the increase of both linoleic and linolenic acids in fly heads. The diet supplied with linseed oil, which contains high amounts of C18:3 and low amounts of C18:2, induced a significant increase of linolenic acid in the fly heads but did not change linoleic acid.

Then, we added single fatty acids to our low-PUFA food source. Those fatty acids were either saturated FA stearic acid (C18:0), or mono-unsaturated FA oleic acid (C18:1), or one of the two PUFAs C18:2 or C18:3. All share similar 18-carbon-long chain and diverge only in the number of unsaturations. Furthermore, all those FAs are commonly found in the fly´s natural food source and make up the great majority of the FA in the two vegetable oils mentioned above. An additional supply of the non-essential fatty acids C18:0 and C18:1 to the low-PUFA diet led to no obvious change in the fatty acid composition of the heads (Fig 1B). However, the total MUFA content in flies raised on low PUFA, low PUFA + C18:0 or, low PUFA + C18:1 was slightly increased in comparison to a diet rich in PUFAs. As presented in Fig 1B, the supply of C18:2 or C18:3 in the low-PUFA food rescued the amount of C18:2 or C18:3 in fly heads.

We next wondered whether the PUFA amount in fly heads, which developed on low-PUFA food could be increased when flies were fed with a specific fatty acid only after adulthood. Therefore, we raised *Drosophila* on low-PUFA food until adulthood. Then, we transferred the adults on low-PUFA food supplemented with C18:3 for 48 h. By ingesting this transitory enriched diet the flies could compensate their lack of PUFAs in their heads. This procedure significantly increased the amount of C18:3 (Fig 1B).

### PUFA Deficient Flies Show Deficits in the Visual Guided Positive Phototaxis Test

**Fig 2:**
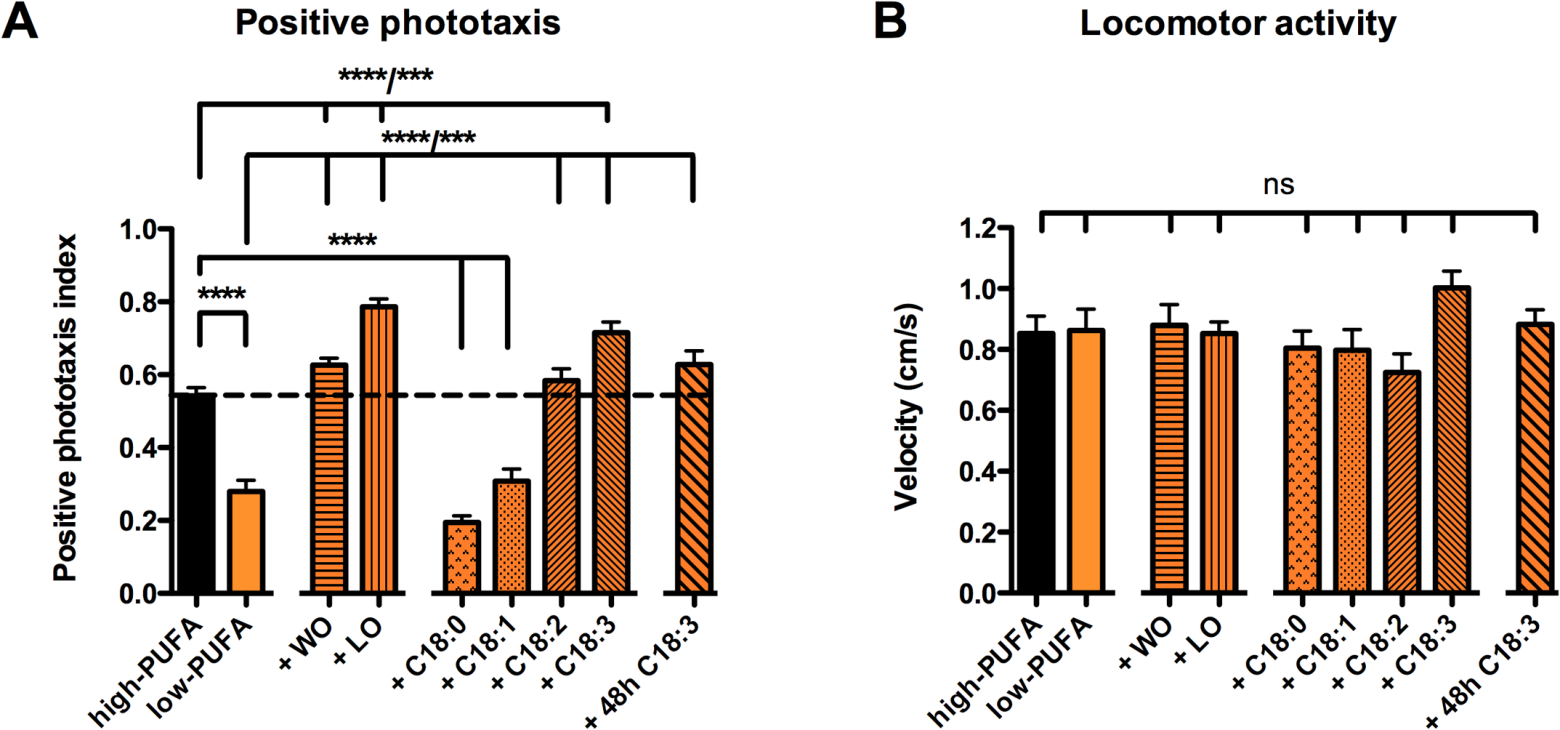
Lack of essential PUFAs leads to a decrease in the visual positive phototaxis behavior. (A) Flies raised on the low-PUFA diet show reduced response to light in the positive phototaxis test when compared to flies which were raised on the high-PUFA diet (*t-test;* ****p < 0.0001). This defect could not be rescued by adding non-essential fatty acids (C18:0 or C18:1) (1-Way-ANOVA; ****p < 0.0001). Adding either oils (WO or LO) that contain both C18:2 and C18:3 in different ratio or single essential PUFAs (C18:2 or C18:3) rescued positive phototactic behavior (1-Way-ANOVA; ****p < 0.0001 or p*** < 0.001, respectively), The observed reduction in phototactic behavior is also rescued when flies were raised on the low-PUFA diet, and then were fed with low-PUFA diet + C18:3 for 48h only during adulthood (KW-test; ***p < 0.001) (N = 25). The significance levels indicate the results of the applied *post-hoc* tests. Detailed information about statistical values can be found in S3A Table (B) The basal locomotory test shows that the visual phenotype observed in A is not due to general locomotory impairments. Data represent mean ± SEM (N = 18, 1-Way-ANOVA or KW-test; ns: *p* > 0.05). Detailed information about statistical values can be found in S3B Table.

After manipulating the amount of PUFAs in the fly heads, we tested the effect of such manipulation on the nervous system function. Therefore, we adapted the well-established fast positive phototaxis test (see Material and Methods). The visual ability to react to light in this test is represented by a positive phototaxis index (PPI). While flies bred on a regular diet had a PPI of 0,54 ± 0,022, flies raised on low-PUFA food showed a significant reduction in their PPI of 0,28 ± 0,031 (*p* < 0,0001; *t-test*, Fig 2A). To test whether this deficit in visual capacities was specific to the absence of PUFAs we first added walnut oil, or linseed oil, to the low-PUFA diet to rescue the levels of C18:2 and C18:3, respectively. Additional supply with either of the two oils rescued a control-like PPI. The addition of linseed oil, which only increased C18:3 in fly heads, even led to an increase in the fly´s phototactic response. We also tested the effect of single fatty acid components for their capacity to rescue the low PPI of PUFA depleted flies. As expected, an additional supply of the non-essential fatty acids, like C18:0 or C18:1 was unable to rescue the low PPI of PUFA-starved flies. In contrast, a full rescue was obtained when adding either C18:2 or C18:3.

Flies raised on the C18:3 enriched diet even showed even an increased PPI in comparison to flies raised on the regular high-PUFA diet. Since we let the tested flies develop on a specific food source, we tested whether the low PPI in PUFA- depleted flies was caused by the absence of PUFAs during preimaginal development or adulthood. To answer this question, we raised flies on low-PUFA food and transferred them for 48h after adult hatching on a low-PUFA diet that contained C18:3. Such shifted flies were able to incorporate C18:3 only during this period of time (Fig 1B) and showed a rescued PPI (Fig 2A). This result strongly suggests that the decreased PPI in PUFA-depleted flies was not caused by a general developmental impairment of their visual system but rather by a transient lack of PUFAs needed for proper signal transmission during adulthood.

Since the phototaxis test depends on the fly’s ability to move, we tested whether the low PPI in PUFA-starved flies was due to defects in visual signal transmission or to a general locomotion impairment. As shown in Fig 2B, no matter on what food source they were raised, all flies showed the same level of locomotor activity. We also tested if the fly’s sensorimotor responses were not affected by the diet by measuring negative geotaxis. Again all animals behaved equally in this assay (S3 Fig). Altogether our behavioral data indicate that flies raised in the absence of PUFAs suffered of specific visual defects. Adding either C18:2 or C18:3, or both PUFAs, in the form of walnut oil or linseed oil enabled us to restore the normal function of the visual system.

### PUFA Deficient Flies Show Deficits in Neuronal Signal Transmission

**Fig 3:**
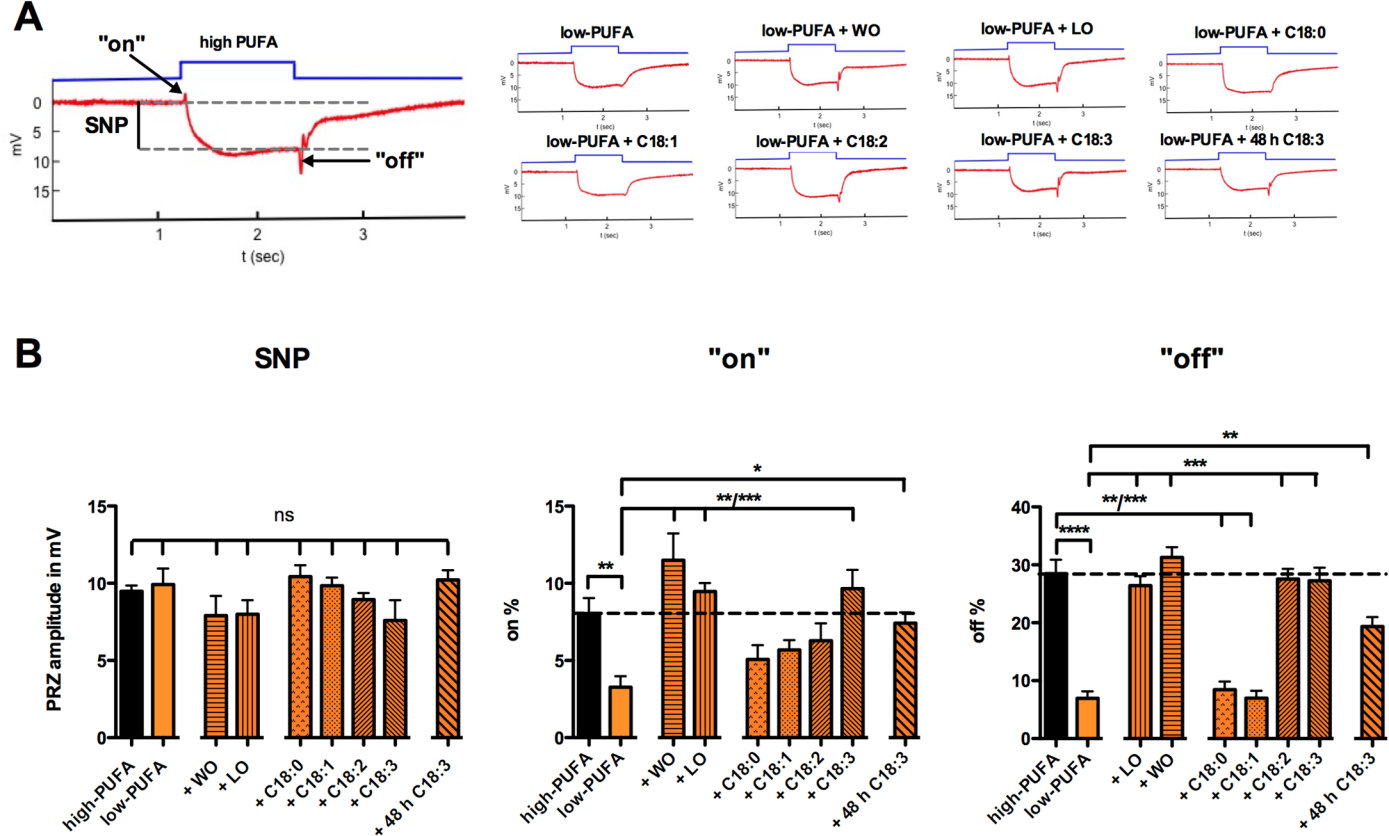
Lack of essential PUFAs causes defects in visual signal transmission. (A) Representative traces of electroretinographic recordings (ERGs). (B) Quantification of the sustained negative potential (SNP) reveals no nutrition-related differences in photoreceptor depolarization. “on”- and “off”- responses were significantly decreased in flies raised on the low-PUFA diet in comparison to flies raised on the high-PUFA diet (Mann-Whitney test; **p < 0.05 or ****p > 0.0001, respectively). The “on”-response could be rescued by adding either C18:3, WO, or LO to the low-PUFA diet (KW-test; **p < 0.01 or ***p < 0.001). Adding either WO, LO, C18:2, or C18:3, to the low-PUFA diet rescued the “off” response (KW-test; ***p < 0.001). Both defects in the “on”- and the “off”-responses of flies raised on the low-PUFA diet could be rescued with 48h of adult diet on high-PUFA + C18:3 (KW-test; p* < 0.05 or p** < 0.01, respectively). Data represent mean ± SEM. (N = 10, ns: *p* > 0.05). The significance levels indicate the results of the applied *post-hoc* tests. Detailed information about statistical values can be found in table ST3C, D and, E.

Our data indicated that PUFA-starved flies suffered specific deficits in the fast positive phototaxis test. However, it remained unknown whether this deficit was caused by the inability of the photoreceptor neurons to depolarize or to transmit the signal to their post-synaptic partners. To answer this question we performed extracellular electroretinographic recordings (ERG) in flies raised on the different diets. During those ERGs the response of the flies’ eyes to light is detected by a recording of sum potentials of the photoreceptors and their postsynaptic partners. The typical ERG trace of a wild-type fly raised on regular high-PUFA diet. It can be dissected into 3 components (Fig 3A). The sustained negative potential (SNP) reflects the depolarization of photoreceptors during a light stimulus. The “on”- and “off”-transients appear at the beginning and at the end of the light stimulus respectively and reflect the proper signal transmission from photoreceptor neurons to their postsynaptic partners [28,33,34]. The photoreceptors of flies raised on the low-PUFA diet depolarized normally, as illustrated by normal SNP amplitude. On the contrary, their ERGs revealed a decrease in their “on”- and “off”-transients indicating defects in signal transmission from photoreceptor cells to their postsynaptic partners (Fig 3A and 3B). As it was the case for the phototaxis test, an additional supply with non-essential fatty acids C18:0 and C18:1 did not restore the amplitude of the “on”- and “off”-transients. A rescue of the “on”-transient could be obtained only with additional doses of C18:3, walnut oil, and linseed oil in low-PUFA food. All those diet supplements increased the amount of C18:3 in fly heads (Fig 1B). The “off”-transient was also restored by adding C18:2 alone.

Confirming that the defects in visual signal transmission were not due to developmental problems, we could observe the rescue of both, the “on”- and the “off”-responses when flies were raised in absence of PUFAs and transferred on low PUFA + 18:3 for 48 h during adulthood. Taken together we conclude that the presence of PUFAs is essential for proper signal transmission from photoreceptors to their postsynaptic partners.

## Discussion

In this study we successfully established and tested a low-PUFA diet in *Drosophila*. This diet contained high amounts of SFAs but very low concentrations of PUFAs (Fig 1A). Flies, which developed on this food source, have extremely low levels of PUFAs in their heads. On the contrary, the amount of SFAs remained stable, most likely because these compounds have been metabolized in anabolic pathways or transformed to MUFAs by the activity of delta-9-desaturases like Desat1.

The use of the low-PUFA diet for the rearing of PUFA deficient flies unveiled the requirement of PUFAs for the proper function of the fly’s visual system. We showed by using the positive phototaxis test that PUFA-depleted flies are unable to detect light properly. By means of electrophysiology our data indicate that visual synaptic transmission in PUFA-starved flies was impaired at the first optical synapse.

### PUFAs Are Dispensible for Photoreceptor Depolarization

Photoisomerized rhodopsin in the photoreceptor cells (PRZ) was shown to activate the phospholipase C_â_ (PLC) [35]. The PLC enzyme then hydrolyses phosphatidylinositol bisphosphate (PIP_2_) into 1,4,5-inositol triphosphate (IP_3_), diacylglycerol (DAG), and a proton. The activation of this second messenger cascade leads to the opening of two ion channels, transient receptor potential (TRP) and TRP-like (TRPL), the efflux of calcium and the depolarization of the photoreceptor cells [36–40]. Multiple studies aimed at identifying the TRP and TRPL agonists and DAG or PUFAs remained the strongest candidates[41–49].

DAG contains a glyceride backbone to which two fatty acid chains are covalently bound. Those chains could be SFAs, MUFAs, or PUFAs. For a long time it was believed that after the income of light PUFAs are cleaved of the glyceride backbone of DAG. Those free PUFAs bind directly to TRP and TRPL, induce their activation and lead to the depolarization of photoreceptors [45,47,48].

On the contrary, Delgado et al. found, that the whole DAG is the agonist of TRP. Moreover, they describe 10 different DAG species in *Drosophila* eyes. They examined which of those are present in the flies´ eyes, produced in a light-dependent manner, and though could be linked to visual signal transmission. It turned out that all unsaturated DAGs, and not necessarily those containing PUFAs, accumulate after light exposure and might therefore be the substrates for TRP [46]. Hence, free PUFAs are not needed for photoreceptor depolarization.

The dispensability of dietary PUFAs that are used to synthetize DAG, containing PUFAs for photoreceptor depolarization could also be confirmed in our study. We clearly show that a lack of dietary PUFAs does not affect the SNP amplitude. Moreover, we demonstrated that the absence of PUFAs specifically induces defects in signal transmission between photoreceptors and their postsynaptic partners.

### PUFAs Are Necessary for Signal Transmission at the First Optical Synapse

The first optical synapse in *Drosophila* vision is a tripartite synapse, which involves the presynaptic PRZ, the postsynaptic neurons and glial cells [50]. Photoreceptors sense the income of light, depolarize, and release the neurotransmitter, histamine, into the synaptic cleft [51]. There, histamine binds to the postsynaptically expressed receptor, a histamine-gated chloride channel encoded by *hclA* [52–54]. Glial cells, which are in close proximity to this synapse, are believed to remove histamine from the cleft and to metabolize histamine into an inactive metabolite, carcinine [50,55,56]. The malfunctioning of all three of these cell types has been reported to decrease vision and to lead to blindness [33,34,54]. Our electrophysiological approach does not allow us to attribute the drastic reduction in the size of the “on”- and “off”-transients in PUFA-starved flies to one of those three cell types.

However, several studies in cultured human or mouse cells, as well as the neuromuscular junction of *C*.*elegans*, have shown that PUFAs are essential for proper neurotransmitter release or the recycling of synaptic vesicles after neurotransmitter release [7,57–59]. In detail, the binding of PUFAs to presynaptic syntaxin changes this protein´s conformation. This allows the formation of the ternary SNARE complex, which promotes vesicle fusion and neurotransmitter release (exocytosis) [60–63].

In addition, PUFAs are enriched in synaptic vesicles [64,65]. As in mammals, invertebrate photoreceptors function through graded potentials rather than action potentials leading to a high consumption of the continuously used synaptic vesicles. Synaptic vesicles are very small in diameter (30–40 nm) and their membrane is highly curved. The degree of membrane curvature, and therefore the size of synaptic vesicles, is highly dependent on the composition and the orientation of its membrane lipids. In *C*.*elegans* a lack of specific PUFAs leads to enlarged synaptic vesicles, which causes defects in vesicle recycling (endocytosis) after neurotransmitter release [58]. Therefore, the defects in signal transmission observed in this study might be due to inefficient exocytosis or endocytosis in the photoreceptors.

Another explanation for the observed transmission problems might be the dysfunction of glial cells due to the lack of PUFAs. In the mammalian brain the metabolism of fatty acids, including the elongation and breakdown of PUFAs, takes place in astrocytes [66,67]. We also reported the efficacy of dietary PUFAs in the prevention of glial activation in the rat retina and retinal structure in a model of glaucoma [68]. Several studies on vertebrates have already shown that an imbalance in glial fatty acids can alter neuronal function [68,69]. Moreover, the direct function of glial-derived PUFAs on synaptic transmission is discussed. However, the precise role of astrocyte PUFA metabolism and its secretion on synapse function remains poorly known [70]. A recent *Drosophila* study found that some fatty acid metabolizing genes (for example: Desat1 and CG1998, a putative fatty acid hydroxylase) are also enriched in the glial cells of invertebrates [71]. Therefore, invertebrate glial cells might also support neurons with PUFAs and thereby impact signal transmission.

## Conclusion

We first established an easy and cheap way to breed *Drosophila* on a PUFA-deficient diet, and to manipulate the amounts of some PUFAs such as omega-3- and omega-6-PUFAs. Second, using the fruit fly´s visual system we tested the synaptic properties resulting from the PUFA deficit. Third, we showed that the omega-3-PUFA (C18:3) fully rescued low-PUFA diet related defects, whereas the omega-6-PUFA (C18:2) only partially rescued the visual electrophysiological defect. We believe that this approach may be further used to screen for genes involved in the PUFA uptake, transport and metabolism in *Drosophila* and to study the action of PUFAs on the synapse function.

## Supporting Information

S1 Fig Relative amount of fatty acid contents in fly heads and fly food. **A-I,** Relative amount of fatty acids in fly heads at the age of 2–9 days. (A) Flies were raised on a high-PUFA diet. (B) Flies were raised on a low-PUFA diet. (C-H) Flies were raised on a low-PUFA diet supplemented with either C18:0, C18:1 C18:2, C18:3, walnut oil, or linseed oil. (I) Flies were raised on a low-PUFA diet and transferred on a low-PUFA diet supplemented with C18:3 for 48 h during adulthood. (J) Relative amount of fatty acids in fly heads at the age of 3 weeks. (K,L) Relative amount of fatty acids in fly food.
(PDF)

S2 Fig Absolute amount of fatty acid contents in fly heads. Data represent the average of 3 groups of 30 heads.
(TIF)

S3 Fig Negative geotaxis assay. Flies raised on various food sources have no defects in their sensorimotor responses. The negative geotaxis assay was performed as described before but with minor modifications [1]. Breefly, 2–9 days old male CantonS flies were banged to the bottom of a tube during 10 seconds. and were given the chance to climb to the top of a 15 cm long tube. The climbing index indicates how many flies (%) were able to climb 2 cm within the next 7 seconds. Data represent mean ± SEM. (N = 5 groups of 21–22 flies, ns = *p* > 0.05). [72]
(PDF)

S1 Table Fatty acid contents in walnut oil and linseed oil. (TIF)

S2 Table Absolute amount of fatty acid contents in fly heads (in μg/head). Data represent the average of 3 groups of 30 heads.
(TIF)

S3 Table Statistics. (A-B) Detailed statistical values corresponding to data shown in Fig 2A and 2B. (C-E) Detailed statistical values corresponding to data shown in Fig 3B: SNP, “on”-transient, and “off”-transient.
(XLSX)
