# Supplementary figures and images for: Lack of Dietary Polyunsaturated Fatty Acids Causes Synapse Dysfunction in the Drosophila Visual System
Source: PLoS One. 2015 Aug 26;10(8):e0135353. doi: 10.1371/journal.pone.0135353 (PMC4550417; doi:10.1371/journal.pone.0135353)

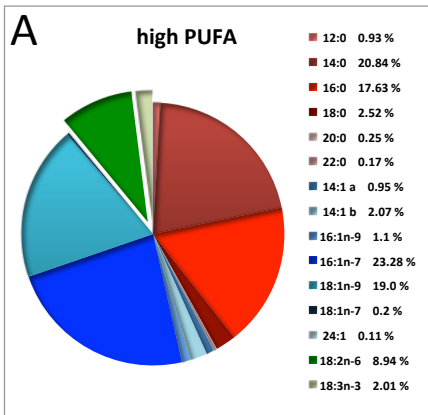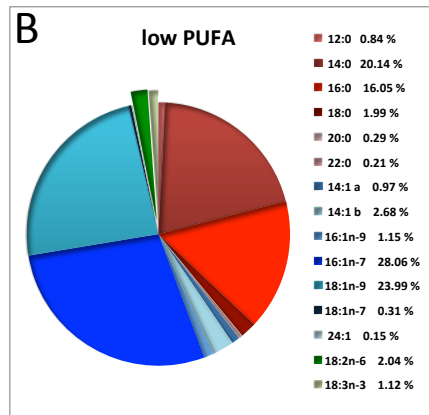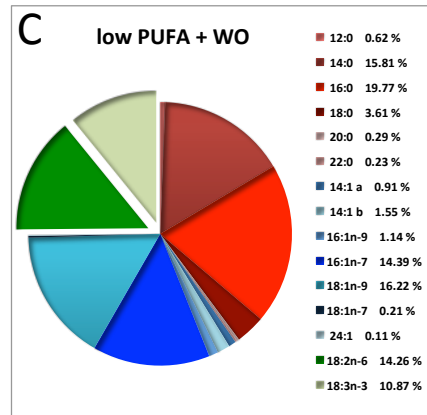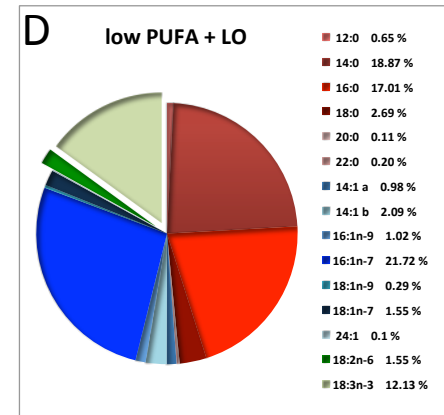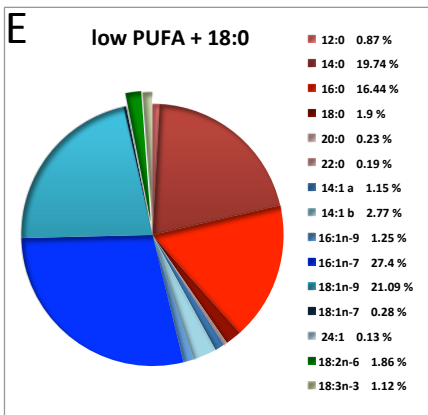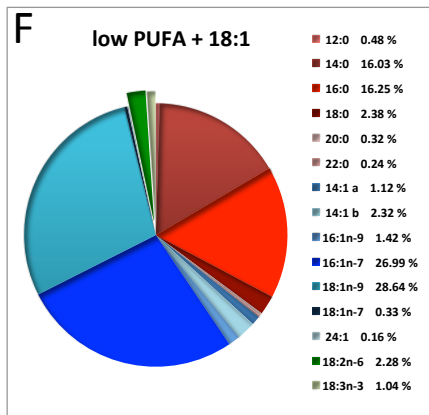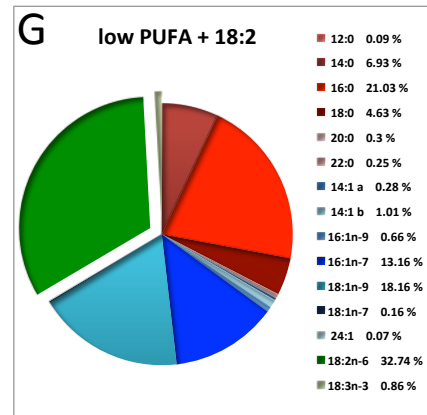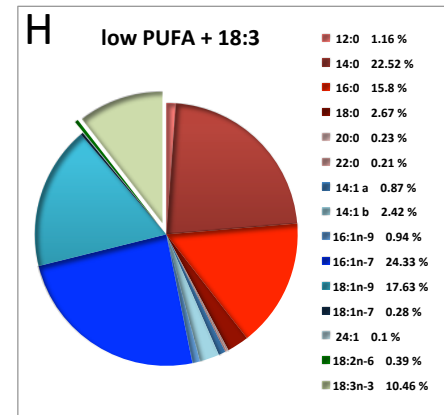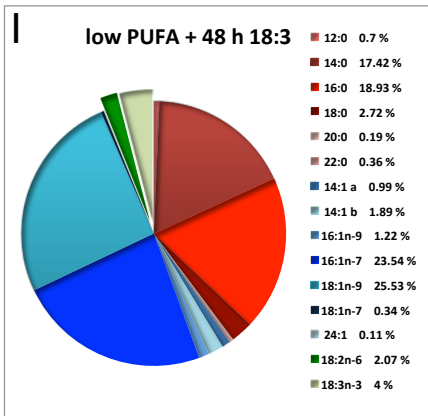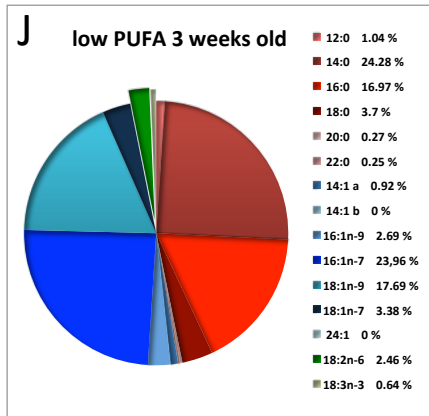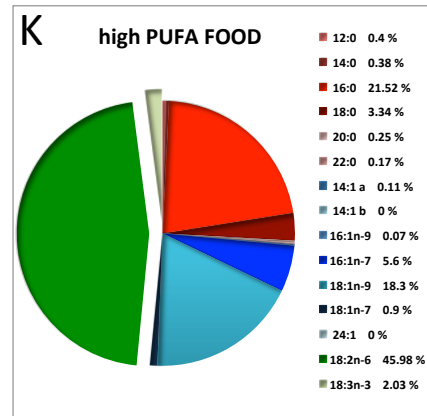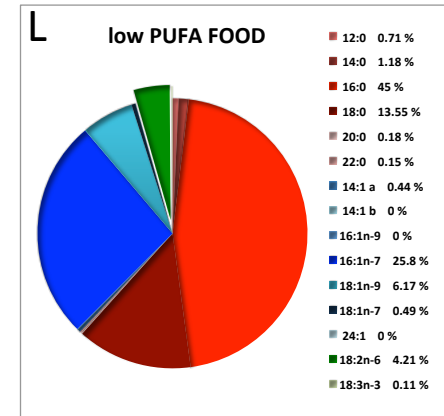

Supplement: S1 Fig — A-I, Relative amount of fatty acids in fly heads at the age of 2–9 days. (A) Flies were raised on a high-PUFA diet. (B) Flies were raised on a low-PUFA diet. (C-H) Flies were raised on a low-PUFA diet supplemented with either C18:0, C18:1 C18:2, C18:3, walnut oil, or linseed oil. (I) Flies were raised on a low-PUFA diet and transferred on a low-PUFA diet supplemented with C18:3 for 48 h during adulthood. (J) Relative amount of fatty acids in fly heads at the age of 3 weeks. (K,L) Relative amount of fatty acids in fly food. (PDF) [file pone.0135353.s001.pdf]

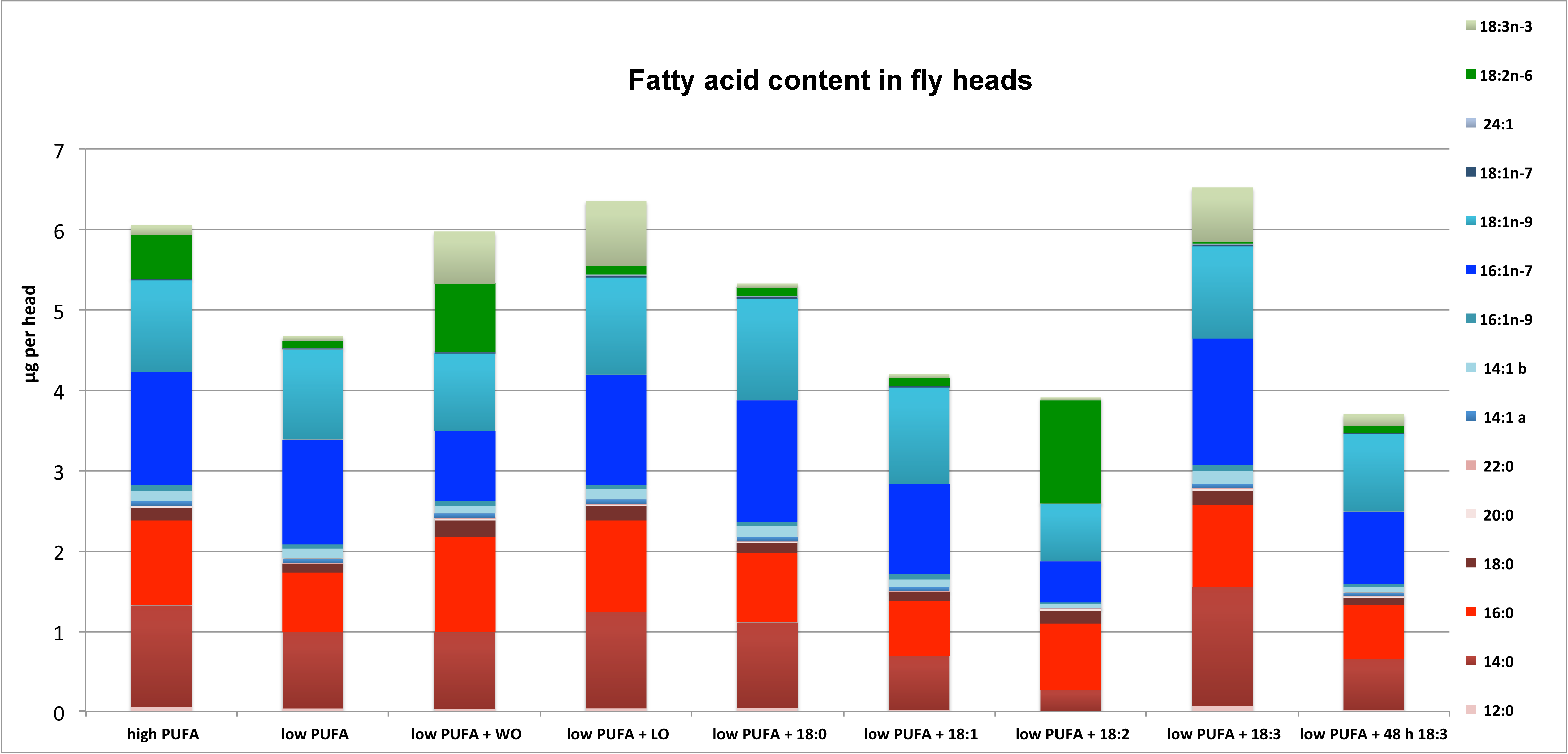

Supplement: S2 Fig — Data represent the average of 3 groups of 30 heads. (TIF) [file pone.0135353.s002.tif]

# negative geotaxis

N = 5

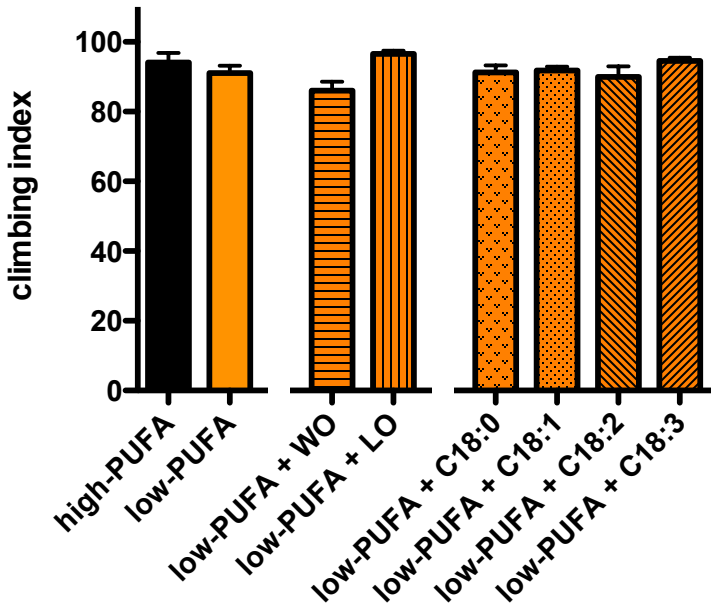

Supplement: S3 Fig — Flies raised on various food sources have no defects in their sensorimotor responses. The negative geotaxis assay was performed as described before but with minor modifications [1]. Breefly, 2–9 days old male CantonS flies were banged to the bottom of a tube during 10 seconds. and were given the chance to climb to the top of a 15 cm long tube. The climbing index indicates how many flies (%) were able to climb 2 cm within the next 7 seconds. Data represent mean ± SEM. (N = 5 groups of 21–22 flies, ns = p > 0.05). [72] (PDF) [file pone.0135353.s003.pdf]

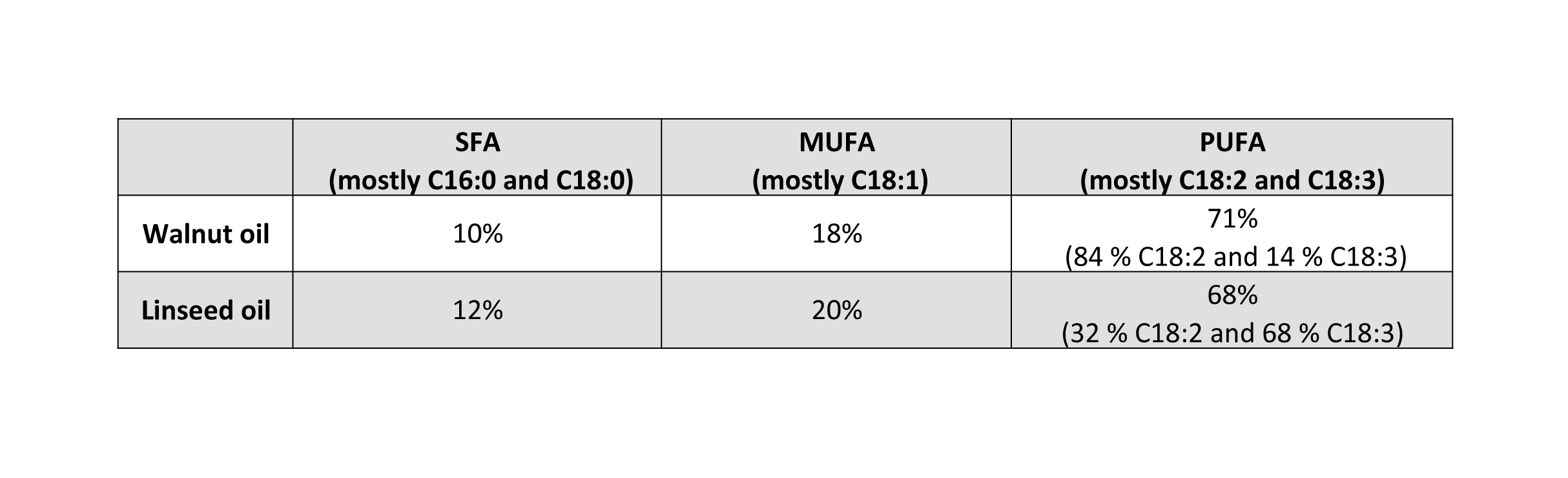

Supplement: S1 Table — (TIF) [file pone.0135353.s004.tif]

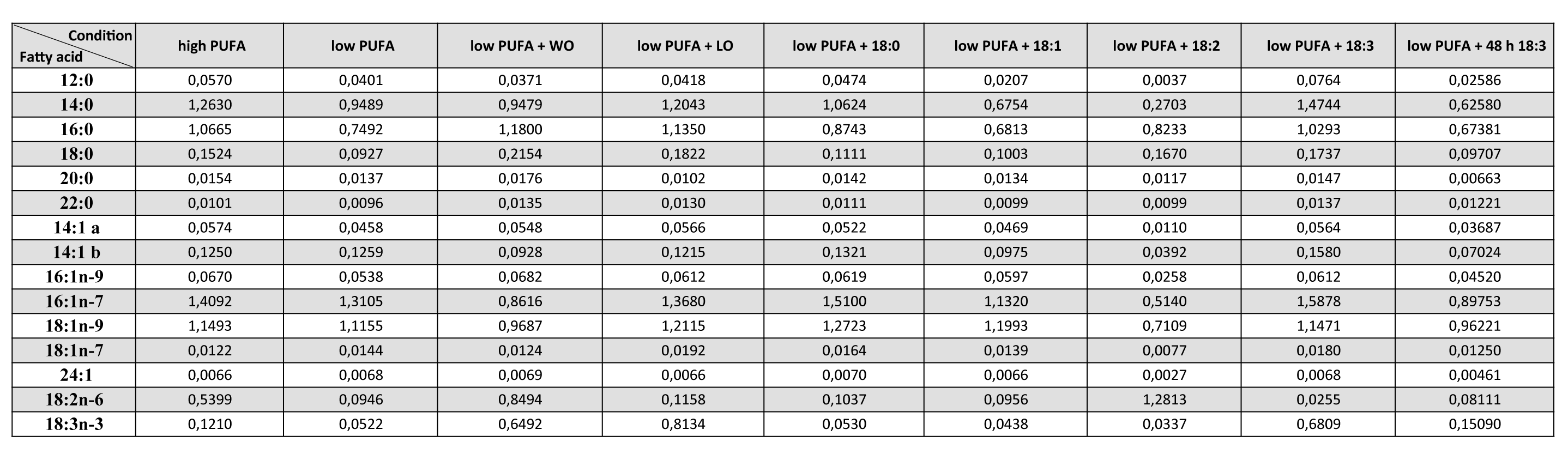

Supplement: S2 Table — Data represent the average of 3 groups of 30 heads. (TIF) [file pone.0135353.s005.tif]
